# Survival of viral pathogens in animal feed ingredients under transboundary shipping models

**DOI:** 10.1371/journal.pone.0194509

**Published:** 2018-03-20

**Authors:** Scott A. Dee, Fernando V. Bauermann, Megan C. Niederwerder, Aaron Singrey, Travis Clement, Marcelo de Lima, Craig Long, Gilbert Patterson, Maureen A. Sheahan, Ana M. M. Stoian, Vlad Petrovan, Cassandra K. Jones, Jon De Jong, Ju Ji, Gordon D. Spronk, Luke Minion, Jane Christopher-Hennings, Jeff J. Zimmerman, Raymond R. R. Rowland, Eric Nelson, Paul Sundberg, Diego G. Diel

**Affiliations:** 1 Pipestone Applied Research, Pipestone Veterinary Services, Pipestone, Minnesota, United States of America; 2 Animal Disease Research and Diagnostic Laboratory, Department of Veterinary and Biomedical Sciences, South Dakota State University, Brookings, South Dakota, United States of America; 3 Department of Diagnostic Medicine/Pathobiology, College of Veterinary Medicine, Kansas State University, Manhattan, Kansas, United States of America; 4 Kansas State Veterinary Diagnostic Laboratory, Kansas State University, Manhattan, Kansas, United States of America; 5 Faculdade de Veterinaria, Universidade Federal de Pelotas, Rio Grande do Sul, Brazil; 6 Center for Animal Health in Appalachia, Lincoln Memorial University, Harrogate, Tennessee, United States of America; 7 Department of Animal Sciences and Industry, College of Agriculture, Kansas State University, Manhattan, Kansas, United States of America; 8 Department of Statistics, College of Liberal Arts and Sciences, Iowa State University, Ames, Iowa, United States of America; 9 Veterinary Diagnostic Population Animal Medicine, Iowa State University, Ames, Iowa, United States of America; 10 Swine Health Information Center, Ames, Iowa, United States of America; Sun Yat-Sen University, CHINA

## Abstract

The goal of this study was to evaluate survival of important viral pathogens of livestock in animal feed ingredients imported daily into the United States under simulated transboundary conditions. Eleven viruses were selected based on global significance and impact to the livestock industry, including Foot and Mouth Disease Virus (FMDV), Classical Swine Fever Virus (CSFV), African Swine Fever Virus (ASFV), Influenza A Virus of Swine (IAV-S), Pseudorabies virus (PRV), Nipah Virus (NiV), Porcine Reproductive and Respiratory Syndrome Virus (PRRSV), Swine Vesicular Disease Virus (SVDV), Vesicular Stomatitis Virus (VSV), Porcine Circovirus Type 2 (PCV2) and Vesicular Exanthema of Swine Virus (VESV). Surrogate viruses with similar genetic and physical properties were used for 6 viruses. Surrogates belonged to the same virus families as target pathogens, and included Senecavirus A (SVA) for FMDV, Bovine Viral Diarrhea Virus (BVDV) for CSFV, Bovine Herpesvirus Type 1 (BHV-1) for PRV, Canine Distemper Virus (CDV) for NiV, Porcine Sapelovirus (PSV) for SVDV and Feline Calicivirus (FCV) for VESV. For the remaining target viruses, actual pathogens were used. Virus survival was evaluated using Trans-Pacific or Trans-Atlantic transboundary models involving representative feed ingredients, transport times and environmental conditions, with samples tested by PCR, VI and/or swine bioassay. SVA (representing FMDV), FCV (representing VESV), BHV-1 (representing PRV), PRRSV, PSV (representing SVDV), ASFV and PCV2 maintained infectivity during transport, while BVDV (representing CSFV), VSV, CDV (representing NiV) and IAV-S did not. Notably, more viruses survived in conventional soybean meal, lysine hydrochloride, choline chloride, vitamin D and pork sausage casings. These results support published data on transboundary risk of PEDV in feed, demonstrate survival of certain viruses in specific feed ingredients (“high-risk combinations”) under conditions simulating transport between continents and provide further evidence that contaminated feed ingredients may represent a risk for transport of pathogens at domestic and global levels.

## Introduction

Historically, the impact of foreign animal diseases (FADs) on global livestock production and economics has been devastating [1]. In 1997, Taiwan and the Netherlands experienced outbreaks of Foot and Mouth Disease (FMD) and Classical Swine Fever (CSF), respectively [2, 3]. In Taiwan, the estimated cost of the FMD outbreak was $379 million, due to the slaughter of over 4 million pigs, approximately 40% of the country’s pig population at the time [2, 4]. In addition, $1.6 billion was lost due to a trade ban of pork to Japan [4]. In the Netherlands, the CSF outbreak resulted in the slaughter of 700,000 pigs across 429 infected farms and the pre-emptive depopulation of 1.1 million pigs from an additional 1300 farms [3]. In 2001, the FMD outbreak in the United Kingdom resulted in the slaughter of 7 million animals, with an overall impact of $11.9-$18.4 billion, including a $4.8 billion loss to agriculture, the food industry and the public sector, $4.2-$4.9 billion in losses to the tourism sector and an additional $2.9-$3.4 billion in indirect losses [5].

While the US has remained free of FMD and CSF over the past several decades, projected losses should an outbreak occur in the country range between $12.9-$14 billion for FMD and $2.6-$9.6 billion for CSF [6, 7]. In addition, the estimated impact of the introduction of African Swine Fever Virus (ASFV) to the US would cost $16.5 billion during the first year of the outbreak [8]. ASFV is a highly contagious pathogen that threatens the swine industry worldwide [9]. Its recent introduction to the Caucasus region and subsequent spread into Eastern Europe, the lack of an effective vaccine, and the role of wild boars and soft ticks in transmission and maintenance of the virus underscores the significance of ASFV and the challenges to disease control [9].

The introduction of Porcine Epidemic Diarrhea Virus (PEDV) into the US in 2013 serves as an example of the impact that exotic diseases may have on the US livestock industry [10]. Although PED is not an OIE notifiable disease, the introduction of the virus into the US resulted in the loss of approximately 7 million pigs or 10% of the annual pig population [11]. The root cause of PEDV introduction to the US has not been conclusively determined; however, contaminated feed and feed ingredients may have served as vehicles for PEDV introduction, as PEDV transmission though contaminated feed has been well documented [12]. Furthermore, given the fact that the original PEDV strain detected in the US shared 99.7–99.8% nucleotide identity with a Chinese PEDV strain, actively circulating in China raised the question of whether contaminated feed could have served as a vehicle for the initial virus entry into the US swine population [13]. By using a Trans-Pacific transportation model we evaluated the possibility of PEDV surviving a trip from China to the US [14]. By spiking feed ingredients commonly imported from China to the US with PEDV, and subjecting the mixtures to environmental conditions simulating a 37-day trip from Beijing to Des Moines, IA [12, 14, 15], we showed that PEDV survived the transport period in five key ingredients used to formulate porcine rations, including soybean meal (organic and conventional), vitamin D, lysine hydrochloride and choline chloride [14].

These results raised important questions as to whether contaminated animal feed and feed ingredients could serve as vehicles for the spread of other viral diseases between countries. Therefore, the objective of this study was to evaluate the survival of select viral pathogens in contaminated feed ingredients using models designed to simulate transportation conditions across different regions of the world. The study was based on the hypothesis that virus survival under the simulated transportation conditions would depend on the combination of virus and ingredient, with some ingredients providing the ideal matrix for survival of select viruses (high-risk combinations).

## Materials and methods

### Selection of viruses and corresponding viral surrogates

The Swine Health Information Center (SHIC) has recently compiled the swine disease matrix (http://www.swinehealth.org/swine-disease-matrix/), which consists of a list of important viral pathogens of swine that were ranked by SHIC based on three criteria: 1) likelihood of entry to the US or becoming an emerging disease if already endemic in the US, 2) economic impact on US production, and 3) impact on domestic and international markets. From this list, 11 pathogens were selected for this study: FMDV, CSFV, ASFV, IAV-S, PRV, NiV, PRRSV, SVDV, VSV, PCV2 and VESV.

Since FMDV, CSFV, PRV, NiV, SVDV and VESV are exotic to the US and most are select agents, closely related surrogate viruses with similar genetic and physicochemical properties were selected (Table 1). For FMDV, the picornavirus Senecavirus A (SVA) was used, while Bovine Viral Diarrhea Virus (BVDV), Bovine Herpesvirus Type 1 (BHV-1), Canine Distemper Virus (CDV), Porcine Sapelovirus (PSV) and Feline Calicivirus, were used as surrogates for CSFV, PRV, NiV, SVDV and VESV, respectively. Surrogate viruses are commonly used to study different aspects of foreign animal disease agents, including studies addressing the environmental stability and the efficacy of disinfectants against these viruses [16–23]. To ensure that surrogate viruses accurately represented target viruses, selection criteria required that both the target and the surrogate were classified in the same viral family, with the closest available virus within the subfamily and/or genus being selected [24]. For the viruses that are endemic in the US (PRRSV, IAV-S, PCV2, and VSV) and for ASFV, the sole member of the *Asfarviridae* family, the actual pathogens were utilized in this case. All studies with endemic pathogens and viral surrogates were performed in biosafety level 2 (BSL-2) laboratory conditions at South Dakota State University (SDSU), while the studies with ASFV were performed in BSL-3 laboratory conditions at the Biosecurity Research Institute (BRI) at Kansas State University (KSU). A complete list of target viruses, viral surrogates and their properties are presented in Table 1.

**Table 1:** Summary of foreign and endemic animal disease target viruses and their respective surrogates.

FAD target virus	Surrogate virus	Viral Family	Genome	Outer membrane	Size
Foot and Mouth Disease Virus	Seneca Virus A	*Picornaviridae*	ss RNA	Non-Enveloped	25–30 nm
Classical Swine Fever Virus	Bovine Virus Diarrhea Virus	*Flaviviridae*	ss RNA	Enveloped	40–80 nm
Pseudorabies Virus	Bovine Herpesvirus-1	*Herpesviridae*	ds DNA	Enveloped	150–200 nm
Vesicular Exanthema of Swine Virus	Feline Calicivirus	*Caliciviridae*	ss RNA	Non-Enveloped	35–40 nm
Nipah Virus	Canine Distemper Virus	*Paramyxoviridae*	ss RNA	Enveloped	150–200 nm
Swine Vesicular Disease Virus	Porcine Sapelovirus	*Picornaviridae*	ss RNA	Non-Enveloped	25–30 nm
Vesicular Stomatitis Virus	Not applicable*	*Rhabdoviridae*	ss RNA	Enveloped	75 nm x 180 nm
Porcine Reproductive and Respiratory Syndrome Virus	Not applicable*	*Arteriviridae*	ss RNA	Enveloped	45–60 nm
Porcine Circovirus type 2	Not applicable*	*Circoviridae*	ss DNA	Non-Enveloped	10–20 nm
African Swine Fever Virus	Not applicable*	*Asfarviridae*	ds DNA	Enveloped	175–215 nm
Influenza A Virus	Not applicable*	*Orthomyxoviridae*	ss RNA	Enveloped	80–120

* = No surrogate virus used. Actual pathogen was used in these cases.

### Transboundary models

**Fig 1:**
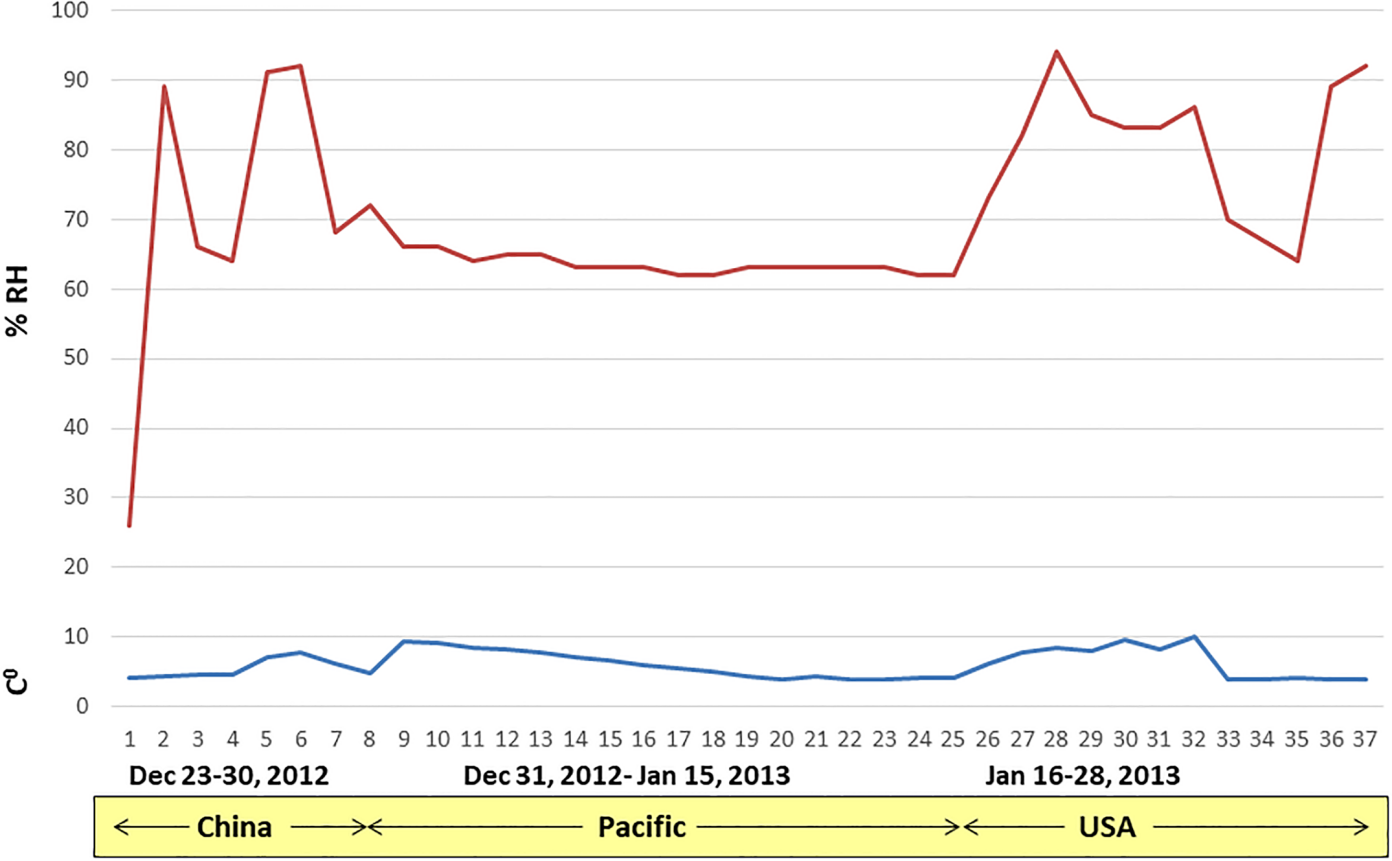
Mean daily temperature and % RH during the Trans-Pacific model. The environmental chamber was programmed to allow for variables to fluctuate several times each day to simulate actual conditions over land and sea.

**Fig 2:**
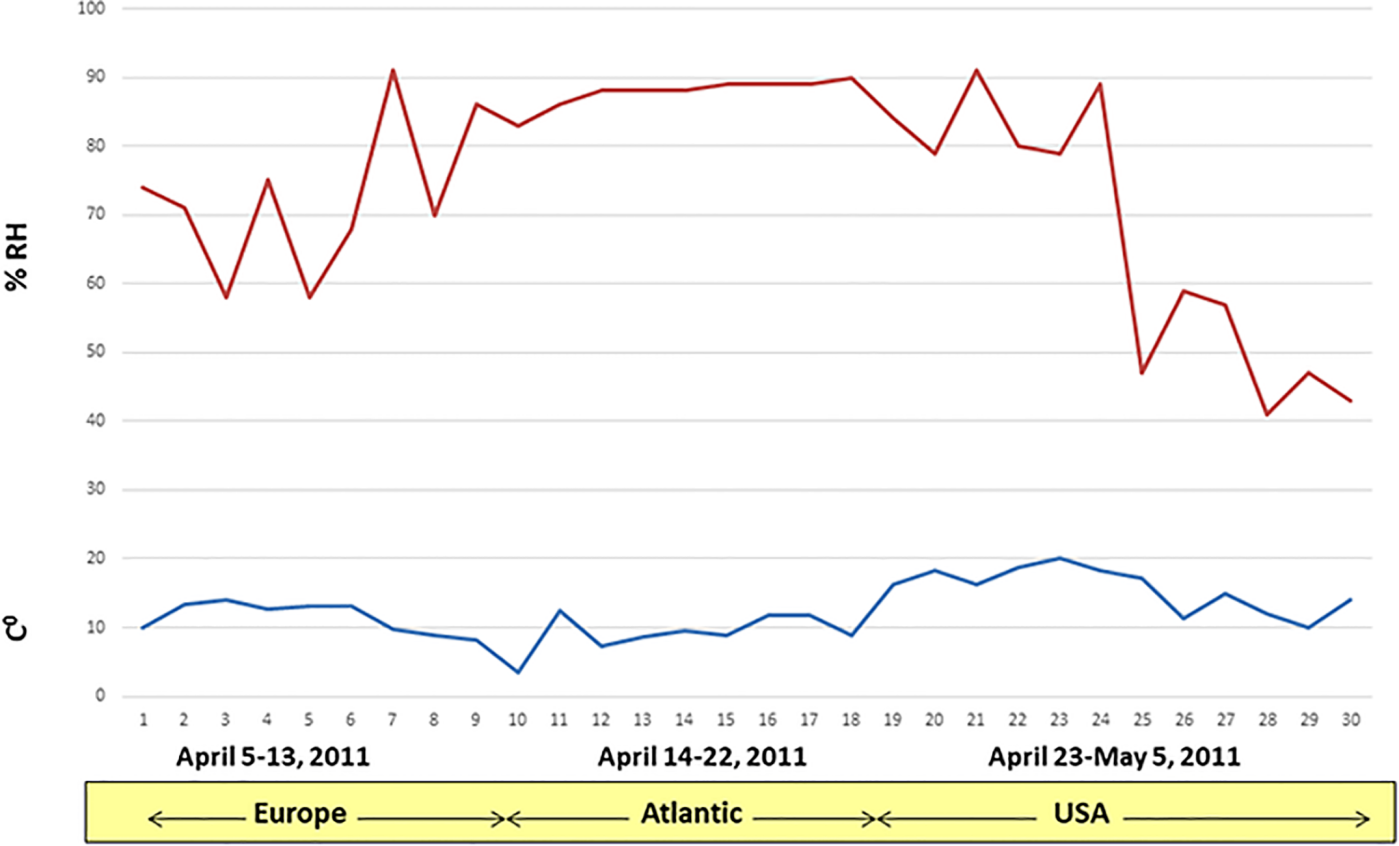
Mean daily temperature and % RH during the Trans-Atlantic model. The environmental chamber was programmed to allow for variables to fluctuate several times each day to simulate actual conditions over land and sea.

To assess the survival of viral pathogens in feed ingredients that are imported into the US, two transboundary shipment/transportation models were developed: a Trans-Pacific model (Fig 1) and a Trans-Atlantic model (Fig 2). The Trans-Pacific model was used to assess the survival of 10 viruses endemic to Asia, more specifically in China, a country from where the US imports tons of animal feed ingredients on a daily basis [14]. The Trans-Atlantic model was used for ASFV, as ASFV has spread throughout the Caucasus, Eastern Europe and the Baltic states during 2007–2015 and is now endemic in the region [9].

#### Trans-Pacific model; timetable and environmental conditions

The website SeaRates.com was used to develop a representative route and timeframe that would model the shipment/transportation of feed ingredients from China to the US [14, 15]. The model assumed that viral contamination of feed ingredients occurred in Beijing, either at the manufacturing plant or post-processing, after which the ingredients would be transported to the Anquing terminal in Shanghai and be held for 7 days in preparation for shipment to the US. The cargo would then be transported across the Pacific Ocean over a 17-day period and enter the US at the port of San Francisco. Following a 7-day period to clear customs, the cargo would then be transported for 2 days via Interstate 80 to Des Moines, IA where it would remain for 3 days. In total, the simulated transport period equaled 37 days.

To simulate environmental conditions (i.e., temperature and relative humidity (RH)) that occur during shipment over land and sea, an environmental curve was generated based on historical temperature and % RH data available for both land segments (China and US; www.wunderground.com), and the oceanic segment of the trip. The data used to generate the environmental curve represented a 37-day trip between Beijing and Des Moines during December 23, 2012 to January 27, 2013. To generate the 37-day environmental curve, available data summarizing temperature and % RH in shipping containers traveling from Asia to the US for the oceanic segments of the model (December 31, 2012 to January 16, 2013) [15] were paired with historical meteorological data for the land segments of the model (December 23–30, 2012 [China], and January 17–27, 2013 [US]). To simulate the effect of daily environmental fluctuations we used temperature data collected at 4 different times of the day (6 AM, 12 PM, 6 PM, 12 AM) and % RH data collected at three different times of the day (8 AM, 12 PM, 4 PM). The environmental curve (Fig 1) was programmed into an environmental chamber and feed ingredients spiked with target viruses were incubated under conditions that mimic real transportation conditions.

#### Trans-Atlantic model; timeframe and environmental conditions

A Trans-Atlantic transportation model was developed to assess the survival of ASFV in feed ingredients. ASFV has been recently reported in Eastern Europe; therefore, the Trans-Atlantic transportation model simulated a westward route from Poland to the US. The model was based on a 30-day transport period (SeaRates.com) and assumed that ASFV contamination of feed ingredients could occur in Warsaw, Poland, either at the manufacturing plant or post-processing, after the ingredients would travel across Western Europe, pass through Hannover, Germany to the port of Le Havre, France, where the cargo would be held for 7 days in preparation for shipment to the US. The cargo would then travel across the Atlantic Ocean over a 9-day period and enter the US at the port of New York City, NY. Following a seven day period to clear customs, it would then be transported for two days via Interstate 80, passing the cities of Cleveland, OH and Chicago, IL to the destination of Des Moines, IA, where it would remain for three days. In total, the transport period equaled 30 days. As with the Trans-Pacific model, an environmental curve consisting of historical temperature and % RH data was generated for both land segments (Europe and US) and the oceanic segment of the trip. The data used to generate the environmental curve represented a 30-day trip between Warsaw and Des Moines during April 5, 2011 to May 4, 2011. Historical temperature and % RH data from shipping containers travelling throughout the North Atlantic during the period of April 14–22, 2011 [25] were combined with meteorological data (www.wunderground.com) for the land segments of the trip (April 4–13 [Europe]; and April 23 to May 4, 2011, [US]), to generate the 30-day environmental curve. To simulate the effect of daily environmental fluctuations, we used temperature and % RH data collected at four different times of the day (6AM, 12 PM, 6 PM, 12AM). The environmental curve (Fig 2) was programmed into an environmental chamber and feed ingredients spiked with ASFV were incubated under conditions that simulate real transportation conditions from Eastern Europe to the US.

### Selection of feed ingredients

Different feed ingredients and/or products of animal origin were used to assess the survival of each target virus. These ingredients or products were selected based on the amount imported into the US (Table 2). This information was obtained at the International Trade Commission Harmonized Tariff Schedule website (www.hs.usitc.gov), which provides a transaction of specific trade commodities between the US and its international trading partners (G. Patterson, personal communication, May 2015 and January 2017). Based on these transactions, a panel of 11 animal feed ingredients and or products of animal origin known to be imported into the US from Asia and/or Eastern Europe were selected. These included organic and conventional soybean meal, soy oil cake, dried distillers' grains with solubles (DDGS), lysine hydrochloride, vitamin D, choline chloride, moist cat food, moist dog food, dry dog food and natural pork sausage casings. The ingredients and products used in our study were obtained from US feed mills or stores, and the same batch of each ingredient was used for all samples across both models. Each ingredient and product were weighted into 5 g duplicate samples that were placed in 50 mL mini bioreactor tubes (Corning Inc., Corning, NY); with vented caps to allow exchange of temperature and humidity between the interior and exterior of the tube. All samples were subjected to gamma irradiation (minimum absorbed dose of 25 kilograys [kGy]; Neutron Products Inc., Dickerson, MD) to eliminate microbial contaminants prior to use. Select ingredients were subjected to bromatological analysis. Ingredients were analyzed at Midwest Laboratories (Omaha, NE, US) and parameters evaluated included protein (crude), fat (crude), fiber (acid detergent), ash, moisture, dry matter, and pH.

**Table 2:** Quantities (kg) of animal feed ingredients imported to San Francisco, US from China between 2012 and 2016.

Ingredient	2012	2013	2014	2015	2016
Soy oil cake	15,126,647	7,977,560	13,545,880	24,201,390	36,962,316
DDGS	4,008,000	2,640,000	2,808,000	2,416,363	1,738,182
Pet food	4,075,353	3,068,722	623,734	51,587	1,412,165
Soybean meal	1,832, 561	1,816,100	1,340,270	979,627	185,400
Pork sausage casings	129,365	216,845	457,427	420,005	582,093
Lysine	33,000	95,000	19,764	2,325,236	2,393,915
Choline	19,000	400	0	0	0
Vitamin D	26,000	21,000	14,000	0	0
**TOTAL (KG)**	**25,249,926**	**7,198,012**	**18,809,075**	**30,394,208**	**43,274,071**

### Sample management

Duplicate samples of each ingredient were organized into four independent batches, with each batch representing a specific sampling point during the Trans-Pacific or Trans-Atlantic model [14]. For the Trans-Pacific model, Batch 1 simulated a set of ingredients representing contamination during product manufacturing and processing in Beijing, China. This set of ingredients was removed from the environmental chamber day 1 post-contamination (DPC) and submitted for testing. Batch 2 simulated ingredients that had been contaminated in Beijing and then had been transported from the manufacturing plant to the Anquing terminal in Shanghai, where they awaited shipment to the US. This batch was submitted for testing at 8 DPC, representing travel time from Beijing to Shanghai and the necessary time in port awaiting shipment. Batch 3 samples simulated the collective time from the Beijing manufacturing plant, time in the Anquing terminal and the time required for the 17-day trans-Pacific shipment to the US terminal of San Francisco, CA. This batch was submitted for testing at 25 DPC. Finally, Batch 4 simulated the total time from manufacturing, trans-Pacific shipment, time in the San Francisco port awaiting customs clearance, followed by transport to Des Moines, IA. This last batch of samples was removed from the environmental chamber and submitted for testing at 37 DPC. For the Trans-Atlantic model, Batch 1 simulated the contamination of ingredients in manufacturing plants in Warsaw, Poland and were tested 1 DPC. Batch 2 simulated transport of ingredients from Warsaw across Western Europe to the Le Havre, France terminal, where they awaited shipment to the US. This batch was tested 8 DPC. Batch 3 simulated the collective period from manufacturing, transportation across Europe and the 9-day trans-Atlantic shipment to the US terminal of New York City, NY, where it awaited customs clearance. This batch was submitted for testing at 17 DPC. Finally, Batch 4 represented the total time from manufacturing, trans-Atlantic shipment, time in the port of New York City, and arrival of feed ingredients at the destination in Des Moines, IA. This final batch was submitted for testing at 30 DPC. As described, at the specific time points listed above, each independent batch of samples was removed from the environmental chamber and processed for testing. This 4-batch approach increased sample size and ensured that all sample containers remained sealed from the time they were inoculated with each virus until the time they were tested, minimizing the risk of cross-contamination.

### Sample inoculation

Five grams of gamma-irradiated ingredients (in 50 mL mini bioreactor tubes) were spiked with 100 μL of MEM (minimum essential media, Gibco, ThermoFisher Scientific, Waltham, MA, US) containing 1 x 10^5^ tissue culture infectious dose 50 (TCID_50_) of each virus [12]. Samples were vortexed for 10 seconds following the addition of the virus inoculum to the feed. Duplicate samples of each ingredient/virus combination were collected on each sampling time point.

### Controls

Five grams of complete swine feed (commercial ration) were inoculated with PBS and used as negative controls in each sampling batch. Five grams of complete swine feed were spiked with 1 x 10^5^ TCID_50_ of each respective virus and used as positive controls. Additionally, stock virus in MEM or RPMI (Roswell Park Memorial Institute media, ThermoFisher Scientific, Waltham, MA, US) was added at the same concentration to an empty 50 mL mini bioreactor tube. The purpose of this control was to determine whether viruses could survive the simulated journey in the absence of a feed matrix. All controls were run in duplicate and were included in all sampling batches.

### Sample incubation and processing

All samples inoculated with the target viruses were incubated in environmental chambers (Model 9005L, Sheldon Manufacturing Inc., Cornelius, OR (Trans-Pacific); Model 3911, Thermo Scientific Forma, Waltham, MA (Trans-Atlantic)) programed to simulate the environmental conditions (temperature and % RH) described above (Trans-Pacific and Trans-Atlantic models). Samples from Batches 1, 2, 3 and 4 were removed from the environmental chamber on appropriate sampling points (described above) and processed for testing by real-time PCR, virus isolation and/or swine bioassays. Each sample was re-suspended in 15 mL sterile PBS, vortexed for 10 seconds and centrifuged. The supernatant was aliquoted and centrifuged for 10,000g for 10 minutes prior to real-time PCR (PCR) and virus isolation. An additional aliquot was stored at -80°C for swine bioassay.

### Diagnostics

#### Polymerase chain reaction (PCR)

All samples from Batches 1 and 4 were tested by real time PCR at the Animal Disease Research and Diagnostic Laboratory (ADRDL) at South Dakota State University or at the Biosecurity Research Institute at Kansas State University in the case of ASFV. These batches were selected to validate successful inoculation and determine the presence of viral nucleic acid at the beginning (Batch 1) and end (Batch 4) of the incubation period. A cycle threshold of ≥40 was considered negative for ASFV and a cycle threshold of ≥38 was considered negative for the remaining viruses.

#### Inoculum preparation and virus isolation

Appropriate cell cultures susceptible to each of the viruses in our study were used to amplify and to assess virus survival in feed samples collected on Batches 1, 2, 3 and 4 by virus isolation. Primary bovine turbinate (BT) cells were used to amplify BVDV-1a strain Singer (NVSL-140-BVDV) and BHV-1 isolate SD1331. SVA strain SD15-26 [26] was amplified in H1299 cells (ATCC^®^ CRL-5803™) while the CDV strain Snyder Hill (ATCC^®^ VR-1587™) and the VSV strain (a recombinant VSV expressing the green fluorescent protein [VSV-GFP] kindly provided by Dr. Asit K. Pattnaik, University of Nebraska-Lincoln School of Veterinary and Biomedical Sciences), were grown in Vero cells (ATCC^®^ CRL-1586™). MARC145 cells were used to amplify PRRSV strain SD-174. Feline Calicivirus strain FCV-2280 (ATCC^®^ CCL-94™) was amplified in CRFK cells (ATCC^®^ VR-2057™). The Swine Influenza virus H1N1 was amplified in MDCK.2 (ATCC^®^ CRL-2936™), while Porcine Sapelovirus strain PS 32 (NVSL 059-PDV) was amplified in ST cells (ATCC^®^ CRL-1746™) and the PCV2 (kindly provided by Dr. Pablo Pineryo, Iowa State University), was amplified in PK15 cells free of PCV1. A splenic homogenate containing ASFV (Georgia 2007/1) was quantified on porcine alveolar macrophages (PAMs). The ASFV Georgia 2007/1 isolate was kindly provided by Linda Dixon at the Pirbright Institute and obtained through the generosity of David Williams at the Commonwealth Scientific and Industrial Research Organization’s Australian Animal Health Laboratory. With exception of H1299 cells (ATCC^®^ CRL-5803™) and PAMs that were cultured using RPMI medium (Corning; ThermoFisher), the cells used for virus amplification, titration and isolation were cultured in minimal essential medium (MEM Corning). Both MEM and RPMI used to culture cells for virus amplification were supplemented with 2 mM of L-glutamine (Corning), 100 U/ml of penicillin (Gibco), 100 μg/mL of streptomycin (Gibco), 50 μg/mL gentamicin sulfate (VWR), 2.5 μg/mL of amphotericin B (Corning), and 5–10% of fetal bovine serum (Hyclone). MDCK cells used for IAV-S amplification and VI were maintained in MEM supplemented with 8% FBS, 5% 5 g/L lactalbumin enzymatic hydrosylate (LAH, BD) and containing 100 U/mL of penicillin (Gibco), 100ug/L of streptomycin (Gibco), 50 ug/mL gentamycin sulfate (VWR) and 2.5 ug/mL of amphotericin B. Replacement media for IAV-S isolation consisted of MEM containing 5% LAH (5g/L) with penicillin/streptomycin, gentamycin and amphotericin B, supplemented with 10 ug/mL trypsin. All cells and fetal bovine serum tested negative for Bovine Viral Diarrhea Virus. For virus propagation, 75-cm^2^ flasks containing 70 to 85% confluent cell monolayers were inoculated with the appropriate virus and incubated at 37°C for 48 to 120 h depending on the virus strain. Following one freeze-thaw cycle, the suspension was centrifuged for 10 min at 1,000 × g. Cell supernatant was collected, aliquot, and stored at −80°C until use. The virus stocks were titrated in 96-well microtiter plates by endpoint dilution. Titers were calculated and expressed as median TCID_50_ [27]. Virus isolation was performed in appropriate cell types cultured in 24, 48, or 96-well plates. The supernatant from feed ingredients processed as described above were inoculated into semi-confluent monolayers. At least three blind passages were performed with each sample. After inoculation, cell cultures were incubated at 37°C with 5% CO2 and monitored for cytopathic effect for 2–6 days. Virus isolation was performed in all samples except for PCV2-spiked samples. All VI procedures were performed at the SDSU ADRDL or at the KSU BRI following standard operating procedures and/or the standards described in the manual of Diagnostic Tests and Vaccine for Terrestrial Animals (http://www.oie.int/manual-of-diagnostic-tests-and-vaccines-for-terrestrial-animals/). Finally, virus titrations were conducted in 96-well plates to determine the amount of viable virus (endpoint titers) present in duplicate samples at the end of the experiment (Batch 4).

### Swine bioassay

#### Facilities and source of animals

All procedures involving animals were reviewed and approved by the SDSU or KSU Institutional Animal Care and Use Committees (SDSU approval numbers: 16-036A and 17-060A; KSU approval number: 3940). All animals were monitored 2–3 times a day by experienced animal care takers under the supervision of SDSU’s and KSU’s veterinarians. Parameters monitored included characteristic clinical signs for each of the target pathogens (i.e. for animals inoculated with viruses targeting the respiratory tract such as IAV-S or PRRSV respiratory signs were monitored more closely). Each animal had its own medical record and supportive treatment consisting of electrolyte solutions and/or anti-inflammatories were administered at the discretion of the attending veterinarians. Animals that were severely sick and were unable to stand/walk or eat and drink were sedated and humanely euthanized (pentobarbital solution). The swine bioassay was used to determine whether viable virus was present in feed ingredient samples that tested positive on PCR but were negative on VI in cell culture. As a porcine model was used, bioassays were performed only on samples inoculated with swine pathogens including SVA, PRRSV, PCV2, PSV, IAV-S and ASFV. Bioassays were conducted under Biosafety Level 2+ conditions at the Animal Resource Wing (ARW) at SDSU [14] or under BSL-3 Ag conditions at the BRI at KSU. Bioassays conducted with endemic viruses or BSL-2 surrogates were performed in four day old piglets that were obtained from a high health swine herd and were tested by the representative PCR tests and serological assays to insure a negative status to the respective pathogen upon arrival. Piglets were housed in stainless steel gnotobiotic units measuring 0.6m W x 1.2m L x 0.6m H. Units were divided into 4 semi-isolated housing units, allowing for 4 piglets per unit with individual feeding arrangements. Ventilation was supplied by an electric fan maintaining sufficient positive pressure inside the canopy to keep the canopy inflated. Incoming and outgoing air to each unit was HEPA-filtered. All incoming and outgoing materials needed during the study (e.g. swabs, injectable medication, bleeding supplies) were passed through an air-tight stainless-steel port and sterilized using 5% peracetic acid before entering or exiting the port.

The ASFV bioassay was conducted with weaned pigs (approximately 21-days old), obtained from a high health commercial source. All pigs were housed in a 66 square meter room and maintained under BSL-3 Ag containment conditions. Pigs were individually housed in 1.8 square meter pens with each pen separated by approximately 2.3 meters. Pens were raised, stainless steel decks with slotted fiberglass flooring. Pens had 3 solid sides with the 4^th^ side consisting of bars and a gate. The room was environmentally controlled and complete exchange of air within the room occurred 14.5 times/hour. To reduce the risk of aerosols in the room, the pens were not sprayed with water during the 5-day post-challenge period.

#### Swine bioassay inoculation

The inocula used for the swine bioassays were prepared as described above. Each piglet was inoculated with 1 mL of the cleared supernatant from processed feed ingredients and monitored for 5–7 days. Piglets inoculated with PRRSV, PCV2 and ASFV were injected via the intramuscular (IM) route, those inoculated with IAV-S were inoculated via the intranasal route (IN), while piglets inoculated with PSV and SVA received the inoculum orally (PO). A negative control piglet (PBS, IM) was included in all bioassays.

#### Piglet sampling and testing

Following inoculation, the health status of each piglet was monitored daily. Blood samples were collected from piglets inoculated with samples spiked with PCV, PRRSV, SVA and ASFV, nasal swabs were collected from piglets inoculated with samples spiked with IAV-S and rectal swabs were collected from piglets inoculated with samples spiked with PSV. Samples were submitted to the SDSU ADRDL and tested by PCR for the specific viruses. Samples from the ASFV bioassay were tested at the KSU BRI. If infection was diagnosed in an animal or a specific unit, all animals were sampled and humanely euthanized.

#### Data analysis

Based on initial inoculation dose and Batch 4 endpoint titers (in units of TCID_50_), half-life (T ½) estimates were calculated. The T ½ analysis was performed by fitting a linear regression model to the data using the SAS 9.4 Proc Reg procedure for each ingredient and virus, using the log of the viral concentration (inoculation dose and endpoint titer) as the response variables and time (initial day and final day) as the explanatory variables. The slope of the respective lines were calculated from these models and half-lives was estimated using -log (2)/slope estimates as previously described [28].

## Results

### Sample size: Ingredients and assays

A total of 1232 samples were tested in the study.

### Detection of nucleic acid

**Fig 3:**
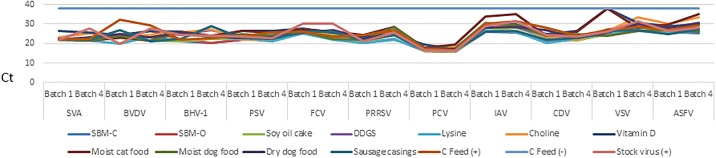
Illustration comparing change in Batch 1 and 4 Ct values across test ingredients and controls.

Of the 1232 samples, 616 were tested by PCR. Fig 3 indicates consistent inoculation and stability of the viral genomes across all ingredients and controls at the Batch 1 and Batch 4 sampling points, respectively, along with PCR-negative results across all negative controls.

### Determination of virus viability

**Fig 4:**
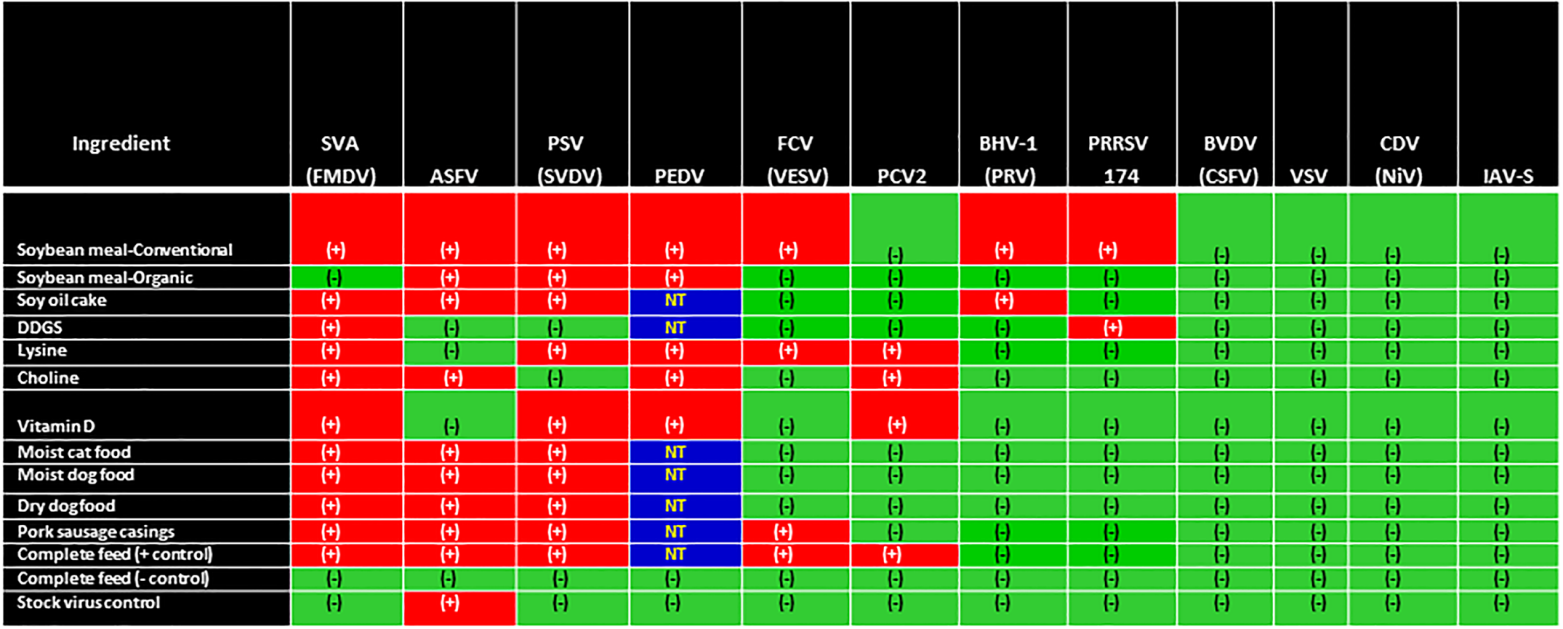
Virus viability in feed ingredient from Batch 4 samples, inclusive of previous PEDV results [14]. A red-colored box with a (+) indicates that virus was recovered in a viable form from a specific ingredient, while a green-colored box with a (-) indicates that viable virus was not recovered by VI and/or swine bioassay. Finally, a blue-colored box with NT denotes that these ingredients were not used in this study and therefore, no results are available.

A total of 1120 virus isolation assays were conducted, involving all viruses except for PCV2, which was tested solely by swine bioassay. A total of 53 animals were used in swine bioassays involving samples spiked with SVA, PRRSV, PSV, PCV2, ASFV and IAV-S. The relationship between virus viability and feed ingredients from Batch 4 samples is provided in Fig 4, inclusive of previous PEDV data [14]. Across the 12 viruses, 7 (SVA, ASFV, PRRSV, PSV, PCV2, FCV, BHV-1 and PEDV) remained viable in 2 or more ingredients; however, a wide variation in viability was observed across viruses. The highest degree of stability was observed for SVA as viable virus was recovered from 10 of the 11 test ingredients, followed by the recovery of viable PSV and ASFV from 9 ingredients, viable FCV and PCV2 from 4 ingredients, and viable PRRSV and BHV-1 from 2 ingredients. In contrast, viable BVDV, VSV, IAV-S and CDV were not detected in any Batch 4 ingredients. Viable SVA, ASFV, FCV, PCV2 and PSV were detected in complete feed positive control samples; however, no evidence of viable virus was detected in complete feed negative control samples nor in the stock virus control samples, with the exception of ASFV which survived the simulated environmental conditions in the absence of a feed matrix (stock virus control). For the Batch 4 samples that were positive via VI (SVA, ASFV, FCV, BHV-1 and PSV), endpoint titers are summarized in Table 3.

**Table 3:** Mean endpoint titers^1^ of viable virus per ingredient type in select Batch 4 samples.

Ingredient	SVA	FCV	BHV-1	PSV	ASFV
**SBM-Conventional**	**1 x 10**^**4.5**^	**1 x 10**^**4.8**^	**1 x 10**^**2.7**^	**1 x 10**^**3.2**^	**1 x 10**^**3.0**^
**SBM-Organic**	neg	neg	neg	**1 x 10**^**3.2**^	**1 x 10**^**3.1**^
**Soy Oil Cake**	**1 x 10**^**3.5**^	neg	**1 x 10**^**2.7**^	**1 x 10**^**3.5**^	**1 x 10**^**3.2**^
**DDGS**	**1 x 10**^**4.3**^	neg	neg	neg	neg
**Lysine**	**1 x 10**^**3.3**^	**1 x 10**^**3.3**^	neg	neg	neg
**Choline**	(+) bioassay	neg	neg	neg	**1 x 10**^**3.2**^
**Vitamin D**	**1 x 10**^**2.3**^	neg	neg	**1 x 10**^**3.5**^	neg
**Moist Cat Food**	**1 x 10**^**4.3**^	neg	neg	**1 x 10**^**3.3**^	**1 x 10**^**3.0**^
**Moist Dog food**	**1 x 10**^**3.3**^	neg	neg	**1 x 10**^**3.7**^	**1 x 10**^**2.8**^
**Dry Dog Food**	**1 x 10**^**3.3**^	neg	neg	**1 x 10**^**3.3**^	**1 x 10**^**2.8**^
**Pork Sausage Casings**	**1 x 10**^**4.3**^	**1 x 10**^**3.7**^	neg	**1 x 10**^**2.8**^	**1 x 10**^**2.9**^
**Complete Feed (+) control**	**1 x 10**^**3.8**^	**1 x 10**^**2.3**^	neg	**1 x 10**^**3.2**^	**1 x 10**^**2.9**^
**Complete Feed (-) control**	neg	neg	neg	neg	neg
**Stock Virus Control**	neg	neg	neg	neg	**1 x 10**^**3.0**^

^1^ = Units of TCID_50_

### Half-life estimates

To determine the rate of decay of virus in feed, half-life estimates were calculated for five pathogens: SVA, PSV, FCV, BHV-1 and ASFV using end point titers determined on Batch 4 samples (Table 4). Overall, half-life appeared to be influenced by virus and ingredient type, with FCV and SVA displaying extended half-lives in samples of conventional soybean meal, 26.6 days and 9.7 days, respectively. SVA appeared to be the most stable virus in feed, with half-lives ranging from 1.7 to 9.7 days across the 10 ingredients in which it survived. Remarkably, FCV presented the longest half-life of all viruses in conventional soybean meal (26.6 days), but its half-life was much shorter (1.9 to 3.5 days) in the other three ingredients that contained viable virus. In contrast, PSV (2.2 to 3.8 days), ASFV (1.3 to 2.2 days) and BHV-1 (2.2 days) displayed shorter, but relatively consistent half-lives across the ingredients in which they survived. Interestingly, the half-life of the ASFV stock virus (1.8 days), was similar to that of virus in the presence of feed matrices.

**Table 4:** An overview of viability across all viruses tested in the study, including PEDV [14] highlighting half-life estimates (in days) of viruses presenting measurable end point titers across ingredients on Batch 4 samples.

INGREDIENT	SVA	ASFV	PSV	PEDV	FCV	PCV2	PRRSV	BHV-1
**SBM-Conventional**	9.7*	1.3*	2.7*		26.6*	•	+	2.2*
**SBM-Organic**	•	1.8*	2.7*		•	•	•	•
**Soy oil cake**	3.4*	2.1*	3.2*		•	•	•	2.2*
**DDGS**	7.1*	•	•		•	•	+	•
**Lysine**	2.6*	•	•		2.8*	+	•	•
**Choline**		2.2*	•		•	+	•	•
**Vitamin D**	1.7*	•	3.5*		•	+	•	•
**Moist cat food**	6.5*	1.3*	3.1*		•	•	•	•
**Moist dog food**	4.1*	1.8*	3.8*		•	•	•	•
**Dry dog food**	2.7*	1.8*	2.8*		•	•	•	•
**Pork sausage casings**	5.6*	1.9*	2.2*		3.5*	•	•	•
**Complete feed (+ control)**	4.1*	1.9*	2.7*		1.9*	+	•	•
**Complete feed (- control)**	•	•	•	•	•	•	•	•
**Stock virus control**	•	1.8*	•	•	•	•	•	•

Note: All ingredients tested for IAV-S, BVDV, CDV, VSV were negative by both VI and bioassay.

* = Endpoint titer T ½ estimate (in days) expressed in units of TCID50/mL

**•** = Negative by both VI and bioassay

^+^ = Negative by VI and positive by bioassay

Light grey shading = While viable PEDV was recovered from these samples, viral titers were expressed in units of FFN, not TCID50

Dark grey shading = Feed ingredients not included in this study

### Relationship of ingredient and virus survival

Ingredients that supported virus survival at a high frequency included conventional soybean meal (*n* = 7), lysine hydrochloride and complete feed (*n* = 5), vitamin D, choline chloride, and sausage casings (*n* = 4), organic soybean meal, the three types of pet food (*n* = 3) and DDGS (*n* = 2). To determine whether specific characteristics of those ingredients could be associated with virus survival, a complete bromatological analysis was conducted on samples of choline chloride, vitamin D, lysine hydrochloride, soy oil cake, DDGS, dry dog food and conventional and organic soybean meal (Table 5). Overall, high levels of crude fat were observed in organic soybean meal, soy oil cake and dry dog food (7%, 9% and 13%, respectively) as compared to the other ingredients tested. In contrast, the highest levels of crude protein were observed in lysine hydrochloride and conventional soybean meal (95% and 46%, respectively). Conventional soybean meal also displayed the highest level of moisture at 12%. Finally, to evaluate whether bromatological characteristics varied by batch or if irradiation had any effect on the bromatological characteristics of the ingredients used in the study, additional samples of several ingredients, including conventional and organic soybean meal, complete feed, lysine hydrochloride, vitamin D, DDGS, and choline chloride were submitted for analysis pre- and post-irradiation (Table 5). Based on similar parameters detected pre- and post-irradiation, it was concluded that irradiation had no negative effect on nutrient characteristics and that results were consistent across batches.

**Table 5:** Bromatological analysis of select ingredients used in the study.

metric	choline	vitamin D	lysine	dry dog food	soy oil cake	SBM-C	SBM-O	DDGS
**pH**	**6.5 (6.4)**	**5.4 (5.2)**	**6.2 (6.2)**	**6.0**	**7.0**	**6.8 (7.0)**	**6.8 (6.9)**	**4.5 (4.6)**
**% moisture**	**6 (7)**	**3 (3)**	**1 (2)**	**8**	**6**	**12 (11)**	**7 (8)**	**11 (12)**
**% dry matter**	**93 (93)**	**97 (98)**	**99 (98)**	**92**	**94**	**88 (89)**	**93 (92)**	**89 (88)**
**% crude protein**	**37 (38)**	**13 (13)**	**95 (95)**	**23**	**45**	**46 (47)**	**44 (45)**	**28 (28)**
**% crude fat**	**0.3 (0.4)**	**0.1 (0.3)**	**0.2 (0.3)**	**13**	**9**	**2 (1)**	**7 (8)**	**7 (8)**
**% fiber**	**14**	**nd**	**nd**	**3**	**7**	**3 (4)**	**4 (6)**	**11 (10)**
**% ash**	**2**	**1**	**nd**	**6**	**6**	**6**	**6**	**5**

nd = not detected, SBM-C = conventional soybean meal, SBM-O = organic soybean meal

Values in parentheses are from irradiated samples and different batches

## Discussion

The objective of this study was to evaluate the ability of important viral pathogens of livestock to survive in animal feed ingredients or feed products frequently imported into the US. To address this, feed ingredients were spiked with viral pathogens or viral surrogates and subjected to incubation under environmental conditions simulating transboundary shipment from Asia or Eastern Europe to the US. These results demonstrate the survival of several viral pathogens in multiple feed ingredients or feed products and confirm our previous findings on the survival of PEDV in feed [14, 29]. Together these findings support the hypothesis that contaminated feed ingredients could serve as vehicles for the transport of viral pathogens between regions, countries or even across continents. Most importantly, our findings expand the scope and highlight the need for improved “feed biosecurity” on imported products that are intended for use in animal diets. Specific conclusions drawn from this project include the following:

1. Viruses can survive in feed, but survival is variable and depends on specific properties of each virus.
2. Certain feed ingredients or feed products present a better matrix for virus survival than others.
3. Select ingredient matrices seemed to enhance the survival of multiple viruses.

As it pertains to the first conclusion, survival clearly differed across viruses. While viable SVA, PSV and ASFV were recovered from most of the ingredients tested, SVA appeared to be the most stable virus in feed. In contrast, while FCV was recovered from only 4 ingredients, its half-life in conventional soybean meal was much longer when compared to SVA and PSV (Table 4). The ability of these select viruses to survive in feed may have been due to their specific structural characteristics, i.e., non-enveloped [24]. Regarding ASFV, while viable virus was recovered from 9 ingredients at the end of the 30-day period, half-lives were relatively short and literally equal to the stock virus control. As its environmental stability is well documented [30, 31], our data support the notion that ASFV survives well, even in the absence of a protective feed matrix. However, it should be noted that ASFV was the only virus tested in the Trans-Atlantic model, which was 7 days shorter than the Trans-Pacific Model. The fact that the shorter incubation of ASFV in feed may account for the apparently higher stability of the virus cannot be formally excluded.

In contrast, several enveloped viruses, such as IAV-S, VSV, CDV, and BVDV did not survive the 37-day transport period. The difference in stability of enveloped and non-enveloped viruses is well documented [32, 33], and our data corroborates with the notion that non-enveloped viruses are more resistant in the environment. Surprisingly, PRRSV, an enveloped virus known to be sensitive to desiccation [34], survived the 37-day transport period in 2 ingredients: conventional soybean meal and DDGS. Although no viable virus was recovered in cell culture, two independent swine bioassays confirmed the survival of the virus in these two ingredients. These findings raise questions as to whether contaminated feed ingredients, such as soy-based or corn-based products could play a role in area spread of PRRSV at the domestic level, or may have played a role in spreading PRRSV throughout the global swine industry.

As stated in the second conclusion, while certain ingredients supported virus survival, others did not. Perhaps the most striking example was the difference in survival between conventional soybean meal versus the organic variety. One possible explanation could be the low fat content of conventional meal in contrast to that present in the organic variety (Table 5), as virucidal effects of medium chain fatty acid blends have been described [35]. However, given the small dataset analyzed in the present study, no definitive conclusions can be drawn regarding the level of fat and/or fatty acids that affect virus survival in these ingredients. Further studies designed to identify nutritional components or processing methods that inhibit virus survival in organic soybean meal could be helpful in improving feed biosecurity.

Finally, the third conclusion is based on the fact that with the exception of ASFV, most viruses (11 of 12) did not survive in the absence of a feed matrix (stock virus controls). These stock virus controls were included in the experimental design to assess whether a virus could survive under the simulated environmental conditions used in the study in the absence of a feed matrix. It is important to note that both models involved moderate temperatures (4–14°C during the Trans-Pacific model and 10–20°C during the Trans-Atlantic model) and broad relative humidity levels (25–95% RH Trans-Pacific and 40–90% RH Trans-Atlantic) which fluctuated daily. The fact that 11 of 12 viruses did not survive these conditions when deposited directly into polypropylene tubes but survived in the presence of feed ingredients suggest that the feed matrix may have provided protection from the environmental conditions encountered during the simulated transport.

Upon careful critique of our study, it is evident that the experimental design presented several strengths. It used rigorous transport models, capable of simulating both Trans-Pacific and Trans-Atlantic shipments of cargo, representative timelines, shipping routes, environmental conditions and ingredients, along with samples that were repeatedly tested using complementary assays. While originally designed to use surrogate viruses, our study assessed the survival of the actual target pathogen in 6 of 12 cases, enhancing the significance of the results. This is evidenced with results demonstrating the survival of ASFV, one of the most significant threats to the swine industry worldwide. While it was not possible to work with pathogens such as FMDV or SVDV, we selected the surrogates SVA and PSV, which are important pathogens in their own right that are closely related to the target pathogens by viral family, presenting similar structure, morphological features and physicochemical properties. In addition, the calculation of half-life was important for several reasons, as it provided a means to compare viral survival across feed ingredients, is independent of viral inoculation dose, and may help assist with biosecurity decision-making, specifically the proper storage time necessary to minimize viral survival in imported ingredients. Finally, the inclusion of multiple controls, including complete feed positive controls, complete feed negative controls and stock virus controls as described earlier, also strengthen our experimental design.

The limitations of the study also need to be discussed. First, it is important to mention that the study used models performed under controlled laboratory conditions which required the use of surrogate viruses in certain cases; therefore, we do not know if FMDV, CSFV, or PRV, for example, would survive the journey, if subjected to these transportation models. Because it was a model, the study was performed in small scale, using volumes of feed that were manageable under laboratory conditions and certainly do not represent real world situations. In addition, the sample sizes used across ingredients tested was small, resulting in insufficient data points to calculate confidence intervals around the half-life estimates. Furthermore, all samples were spiked with an equal amount of virus, which may or may not reflect the actual level of contamination that would occur in the field. As this information is currently unavailable for all target pathogens, we relied on data from viral load in feed from field cases of PEDV [12]. Furthermore, while we demonstrated the infectivity of PEDV, PSV and SVA to piglets via the oral route in the bioassay procedure, with the exception of PEDV, we do not currently know the oral infectious dose of these viral pathogens in feed. Finally, the study needs to be extensively replicated across multiple, independent laboratories, ideally using actual pathogens such as FMDV, CSFV, and PRV to validate and expand upon our results.

In conclusion, this study has provided new information supporting the hypothesis that the “high-risk combinations” i.e. “the right virus paired with the right ingredient”, may be a mechanism for the transboundary transport of pathogens, which is clearly a new paradigm. These data are applicable to viral diseases across many livestock species, including several diseases of swine, ruminants (FMDV, VSV and PRV), avian (IAV-S) and humans (IAV, NiV). In addition, development of the transboundary model provides a platform by which further testing can be performed using other infectious or toxic agents, such as bacteria or chemicals, which will help to build a database regarding the risk of introducing contaminants through feed. In the end, it is hoped that the results of this study will stimulate communication and collaboration between the feed and livestock industries, resulting in further research into the emerging concept of “global feed biosecurity”. Ideally, this new information will enhance the accuracy of risk assessments, promote the development of efficacious feed-based mitigation strategies, and ultimately result in a change in philosophy regarding the global trade of feed ingredients from one that is based on price, to one where country of origin health status is a major consideration.
