# Correction: Survival of viral pathogens in animal feed ingredients under transboundary shipping models

**DOI:** 10.1371/journal.pone.0208130

**Published:** 2018-11-30

**Authors:** Scott A. Dee, Fernando V. Bauermann, Megan C. Niederwerder, Aaron Singrey, Travis Clement, Marcelo de Lima, Craig Long, Gilbert Patterson, Maureen A. Sheahan, Ana M. M. Stoian, Vlad Petrovan, Cassandra K. Jones, Jon De Jong, Ju Ji, Gordon D. Spronk, Luke Minion, Jane Christopher-Hennings, Jeff J. Zimmerman, Raymond R. R. Rowland, Eric Nelson, Paul Sundberg, Diego G. Diel

The data underlying this study were omitted from the original article. The authors have provided the complete dataset below as S1 File and S2 File.

## Supporting information

S1 File Trans-Atlantic environmental curve data. (XLSX)

S2 File Trans-Pacific environmental curve data. (XLSX)
