# Correction: Survival of viral pathogens in animal feed ingredients under transboundary shipping models

**DOI:** 10.1371/journal.pone.0214529

**Published:** 2019-03-21

**Authors:** Scott A. Dee, Fernando V. Bauermann, Megan C. Niederwerder, Aaron Singrey, Travis Clement, Marcelo de Lima, Craig Long, Gilbert Patterson, Maureen A. Sheahan, Ana M. M. Stoian, Vlad Petrovan, Cassandra K. Jones, Jon De Jong, Ju Ji, Gordon D. Spronk, Luke Minion, Jane Christopher-Hennings, Jeff J. Zimmerman, Raymond R. R. Rowland, Eric Nelson, Paul Sundberg, Diego G. Diel

Due to new calculations, there are revisions to the Results section under the sub-heading “Half-life estimates.” Please see the corrected text here:

To determine the rate of decay of virus in feed, half-life estimates were calculated for five pathogens: SVA, PSV, FCV, BHV-1 and ASFV using end point titers determined on Batch 4 samples (Table 4). Overall, half-life appeared to be influenced by virus and ingredient type, with FCV and SVA displaying extended half-lives in samples of conventional soybean meal, 44.4 days and 22.3 days, respectively. SVA appeared to be the most stable virus in feed, with half-lives ranging from 3.9 to 22.3 days across the 10 ingredients in which it survived. Remarkably, FCV presented the longest half-life of all viruses in conventional soybean meal (44.6 days), but its half-life was much shorter (4.3 to 7.9 days) in the other three ingredients that contained viable virus. In contrast, PSV (5.1 to 8.6 days), ASFV (4.1 to 5.1 days) and BHV-1 (4.4 days) displayed shorter, but relatively consistent half-lives across the ingredients in which they survived. Interestingly, the half-life of the ASFV stock virus (4.7 days), was similar to that of virus in the presence of feed matrices.

As a result of the new calculations, there are updates to Table 4. Please see the corrected Table 4 here.

**Table 4:** An overview of viability across all viruses tested in the study, including PEDV [14] highlighting half-life estimates (in days) of viruses presenting measurable endpoint titers across ingredients on Batch 4 samples.

INGREDIENT	SVA	ASFV	PSV	PEDV	FCV	PCV2	PRRSV	BHV-1
**SBM-Conventional**	22.3*	4.6*	6.2*		44.5*	•	**+**	4.4*
**SBM-Organic**	•	4.7*	6.2*		•	•	•	•
**Soy oil cake**	7.4*	5.0*	7.2*		•	•	•	4.4*
**DDGS**	14.8*	•	•		•	•	**+**	•
**Lysine**	5.9*	•	•		6.6*	**+**	•	•
**Choline**	•	5.1*	•		•	**+**	•	•
**Vitamin D**	3.9*	•	7.7*		•	**+**	•	•
**Moist cat food**	14.9*	4.6*	6.7*		•	•	•	•
**Moist dog food**	8.9*	4.2*	8.6*		•	•	•	•
**Dry dog food**	6.4*	4.1*	6.6*		•	•	•	•
**Pork sausage casings**	12.7*	4.4*	5.1*		7.9*	•	•	•
**Complete feed (+ control)**	8.9*	4.3*	6.2*		4.3*	**+**	•	•
**Complete feed (- control)**	•	•	•	•	•	•	•	•
**Stock virus control**	•	4.7*	•	•	•	•	•	•

Note: All ingredients tested for IAV-S, BVDV, CDV, VSV were negative by both VI and bioassay.

* = Endpoint titer T ½estimate (in days) expressed in units of TCID50

• = Negative by both VI and bioassay

+ = Negative by VI and positive by bioassay

Light grey shading = While viable PEDV was recovered from these samples, viral titers were expressed in units of FFU, not TCID50 Dark grey shading = Feed ingredients not included in this study
